# Prospective associations with physiological, psychosocial and educational outcomes of meeting Australian 24-Hour Movement Guidelines for the Early Years

**DOI:** 10.1186/s12966-020-00935-6

**Published:** 2020-03-10

**Authors:** Trina Hinkley, Anna Timperio, Amanda Watson, Rachel L. Duckham, Anthony D. Okely, Dylan Cliff, Alison Carver, Kylie D. Hesketh

**Affiliations:** 1 Institute for Physical Activity and Nutrition (IPAN), School of Exercise and Nutrition Science, Deakin University, 1 Gheringhap Street, Geelong, VIC 3220 Australia; 2 Alliance for Research in Exercise, Nutrition and Activity (ARENA), School of Health Sciences, University of South Australia, Adelaide, Australia; 3 Australian Institute for Musculoskeletal Sciences (AMISS), St. Albans, Melbourne, VIC Australia; 4 Early Start, Faculty of Social Sciences, University of Wollongong, Wollongong, Australia; 5 Illawarra Health and Medical Research Institute, Wollongong, Australia; 6 Mary MacKillop Institute for Health Research, Australian Catholic University, Melbourne, Australia

**Keywords:** Movement behaviours, Physical activity, Sedentary behavior, Sleep, Early childhood, Psychosocial, Physiological, Educational achievement

## Abstract

**Background:**

Several countries have released movement guidelines for children under 5 that incorporate guidelines for sleep, physical activity and sedentary behavior. This study examines prospective associations of preschool children’s compliance with the 24-Hour Australian movement guidelines (sleep, physical activity, screen time) and physiological, psychosocial and educational outcomes during primary school.

**Methods:**

Data were from the Healthy Active Preschool and Primary Years Study (Melbourne, Australia; *n* = 471; 3–5 years; 2008/9). Follow-ups occurred at 3 (2011/12; 6–8 years), 6 (2014/15; 9–11 years) and 7 (2016; 10–12 years) years post baseline. Multiple regression models assessed associations between compliance with guidelines at baseline and later outcomes.

**Results:**

Children were 4.6 years at baseline (53% boys; 62% high socio-economic families). Most children met physical activity (89%) and sleep (93%) guidelines; 23% met screen-time guidelines; and 20% met all guidelines at baseline. Meeting all of the three guidelines was associated with lower BMI z-scores at 9–11 years of age (b = − 0.26, 95%CI -0.47, − 0.05). Meeting physical activity guidelines was associated with higher total body bone mineral density (b = 0.64, 95%CI 0.15, 1.13), and total body bone mineral content (b = 183.19, 95%CI 69.92, 296.46) at 10–12 years of age. Meeting sleep guidelines was associated with better reading (b = 37.60, 95%CI 6.74, 68.46), spelling (b = 34.95, 95%CI 6.65, 63.25), numeracy (b = 39.09, 95%CI 11.75, 66.44), language (b = 44.31, 95%CI 11.77, 76.85) and writing (b = 25.93, 95%CI 0.30, 51.57) at 8–9 years of age. No associations were evident for compliance with screen-time guidelines or for psychosocial outcomes.

**Conclusions:**

Compliance with different movement behavior guidelines was associated with different outcomes. Strategies to support children in meeting all of the guidelines are warranted to maximize health and educational outcomes. Future research investigating dose-response associations, and potential mechanisms, is necessary.

## Introduction

Recent paradigm shifts in guideline development [1–3] have produced 24-Hour integrated movement guidelines. Such guidelines recognize the importance of physical activity (PA), sedentary behavior (SB) – including sedentary screen-time – and sleep. These guidelines should be considered individually and in combination when examining impact on children’s outcomes [4, 5]. Evidence in school-aged populations suggests those engaged in desirable combinations of PA (e.g. high moderate- to vigorous-intensity PA), SB (e.g. low screen-time) and sleep (e.g. high duration) exhibit more beneficial outcomes compared with those engaged in less desirable combinations [5–7]. However, less is known about how preschoolers’ (roughly 3–5 years of age) behaviors [8–11] are associated with future outcomes [12, 13].

Although age ranges vary, recently released Australian (3–5 year olds) and Canadian and World Health Organisation (3–4 year olds) guidelines [2, 3, 14] recommend that a healthy 24-Hour for preschoolers should comprise: ≥180 min of total PA (light-, moderate- and vigorous-intensity PA), with ≥60 min in energetic play (moderate- to vigorous-intensity PA); sedentary screen-time of ≤1 h; and 10–13 h of good quality sleep. The Behavioural Epidemiology Framework suggests that an essential element of determining if a behavior should be targeted for intervention is determining associations between the behavior and health or developmental outcomes [15]. Guidelines were informed by available evidence [2, 16] which was synthesized in a number of systematic reviews [8–11]. Those reviews suggest that health behaviors during the preschool years are associated with multiple outcomes during later years. For instance, physical activity was favorably associated with motor development, fitness, bone and skeletal health [10]. Screen time sedentary behaviors were unfavorably associated with indicators of adiposity, motor or cognitive development, and psychosocial health [11]. Poorer sleep behaviors were associated with higher levels of adiposity, poorer emotional regulation, impaired growth, and higher risk of injuries [9]. Few studies have investigated combinations of behaviors and associations with outcomes [8]. Findings from studies identified suggest that the most ideal combinations of behaviors are favorably associated with motor development and fitness and not associated with adiposity or growth among preschool-aged children [8].

Recent studies suggest compliance with all movement guidelines is low, ranging from 4.3 to 14.9% [17–19] and longitudinal associations with health and developmental outcomes are not well established. Of the few studies in preschoolers which examine multiple behaviors concurrently (both as continuous variables and compliance with guidelines), only 3 have used a prospective design across 1 [20], 2 [21] and 4 [22] year follow-ups. However, those studies have only examined associations with adiposity [20–22] and bone health [22]. Across studies, null associations were reported between behaviors and indicators of adiposity, and small associations between physical activity and indicators of bone health were evident [22].Thus, evidence is lacking of the prospective associations of preschoolers’ guideline compliance with later physiological, psychosocial and educational outcomes. A few studies have recently been published investigating preschoolers’ compliance with the 24 Hour movement guidelines and various outcomes either cross-sectionally or following up to 4 year follow-ups. However, this study is the first to investigate associations of preschoolers’ compliance with those guidelines and associations with physiological, psychosocial and educational outcomes up to 7 years post baseline. Using the Behavioural Epidemiology Framework as a foundation, the aims of this paper were to explore:

1. preschoolers’ compliance with movement (PA, SB and sleep) guidelines;
2. prospective associations between compliance with each guideline and outcomes during primary school; and
3. prospective associations between compliance with all three guidelines and outcomes during primary school.

## Methods

### Study setting and participants

**Fig. 1:**
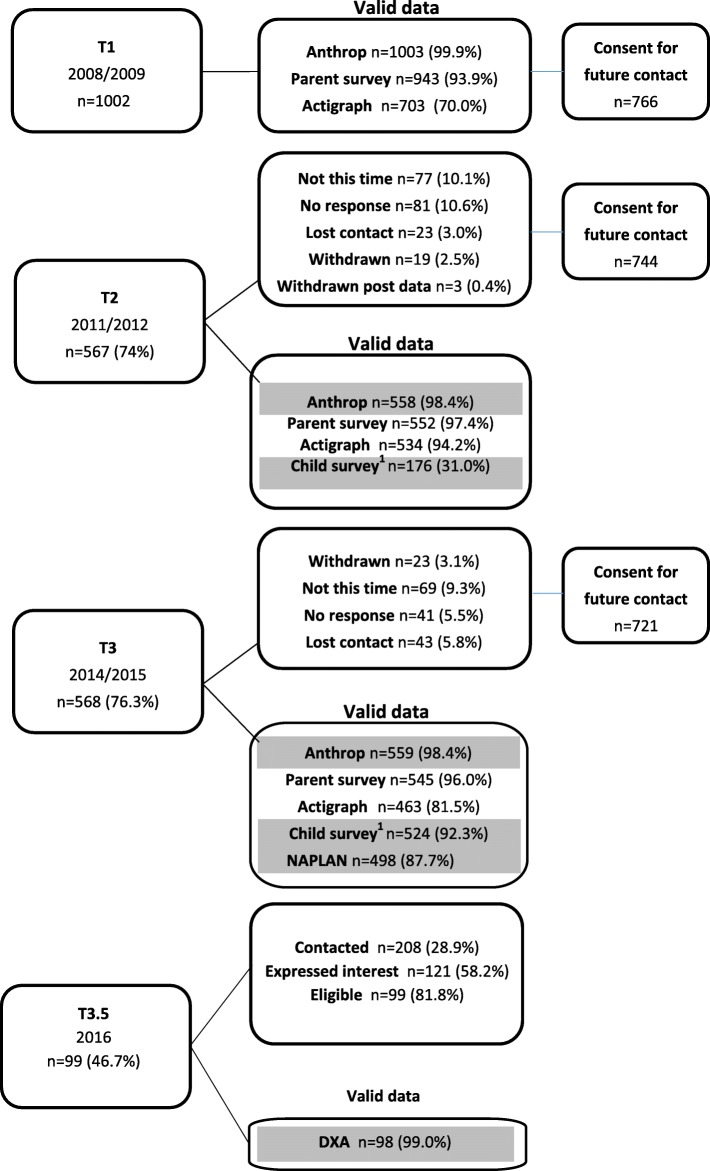
Flow chart of participants through the Healthy Active Preschool Years Study, T1 through T3.5. ^1^T2 Child survey: optional component offered to approximately 60% of the 2009 cohort. Children generally completed 2 of the following: Harter's Self Perception Profile ofr Children (SPPC), BarOn EQ-i:YV or a pictorial scale of perceived movement competence. T3 child survey: SPPC

Data were from the Healthy Active Preschool and Primary Years (HAPPY) Study. Figure 1 presents a flow chart of participants. As previously reported [23], 65 preschools (where children attend for an educational program) and 71 long day-care centers (where children attend for child care which may also include an educational program) participated in the study. This represented 47% of 137 preschools and 46% of 156 long day-care centers invited, in randomly selected low-, mid- and high-socioeconomic areas of metropolitan Melbourne, Australia (2008/9; T1). At baseline, 1002 families (11% of the 9494 invited families) participated, of which 766 (76%) provided consent for re-contact. Children were eligible to participate if they were aged 3–5 years of age and enrolled at a participating center. There were no exclusion criteria. Follow-ups were undertaken in 2011/2 when children were aged 6–8 years (T2: 567 [74%] participated) and 2014/5 when children were aged 9–11 years (T3: 568 [76%] participated). A sub sample of HAPPY participants (*n* = 208) with previous objective measurements of sedentary behaviour from baseline (T1) and at least one other time point (T2 or T3) were identified from the pool of 450 children remaining in the study. Those participants were stratified into tertiles at each time point based on mean sedentary behaviour z-scores. The z-scores were internally generated within the HAPPY sample based on the sample mean and standard deviation at each timepoint. To recruit participants with contrasting sedentary behaviour levels, those consistently (at each time point they provided data) in the top and bottom tertiles for sedentary behaviour were invited to participate in the sub study. This resulted in a sub sample of 99 (girls = 45) (mean age 12.2 + 0.8 years) providing bone outcome data for this paper in 2016 (T3.5).

The Deakin University Human Research Ethics Committee (EC291–2007) and the Victorian Department of Education and Early Childhood Development provided ethical approval for the study. Reporting of this study complies with the STROBE guidelines [24, 25].

### Measures

#### Exposure variables

##### Physical activity (T1)

Children wore ActiGraph GT1M accelerometers (on an elastic belt at the right hip during waking hours, removing for bathing, swimming) for 8 days at T1. ActiGraph accelerometers have established validity, reliability and utility [26]. Data were collected in 15-s epochs [26, 27]. The fourth complete minute of counts above zero after 4 am, with a 1-min (4-epoch) tolerance indicated daily monitoring start-times. Consecutive zero counts for ≥20-min indicated non-wear time; days with > 18-h of data were considered improbable and data removed for that day. Children with data for ≥6 h/day on ≥3 week and ≥ 1 weekend days were included in analyses [28]. A cut-point of ≥25 counts per 15 s epoch identified time in total PA, and ≥ 420 counts per 15 s epoch identified time in moderate- to vigorous-intensity physical activity (MVPA) [29]. Mean time (minutes per day) engaged in total PA, and mean time in MVPA, were calculated. Children met the PA guideline if they had an average of ≥3 h/d of total PA *and* ≥ 1 h/d of MVPA across monitoring time [2, 3].

##### Screen-time (T1)

Parents reported their child’s week (Monday-Friday) and weekend (Saturday-Sunday) sedentary screen-time in TV/DVD/video viewing, internet/computer use and electronic games using a reliable survey [30] at T1. Average daily screen-time was computed and children met the screen-time guideline if this was ≤1 h/d.

##### Sleep (T1)

Parents reported the duration of their child’s usual night- and day-time sleep at T1. Total daily sleep time was calculated, previously shown to provide a reliable estimate of children’s sleep [30]. Children who slept 10-13 h/d on average met the sleep guideline.

##### Compliance with all guidelines (T1)

Children were determined to be compliant with all guidelines if they met each of the three individual guidelines described above.

### Outcome variables

#### Physiological outcomes

##### Adiposity (T1, T2, T3)

At T1, T2 and T3, researchers measured children’s height and weight using Wedderburn Seca portable rigid stadiometers, and Wedderburn Tanita portable digital scales respectively, following standardized measurement procedures [31, 32]. BMI z-scores, adjusted for age and sex [33], were calculated. Waist circumference was measured at the umbilicus at T2 and T3 using a steel, non-stretch tape measure. All measures were taken twice. If measurements differed by > 0.5 cm, > 0.5 kg or > 1.0 cm, respectively, a third measure was taken and the average of the closest 2 used in analyses.

##### Bone measures, density and fractures (T3.5)

Total body (less head) and lumbar spine (L2-L4) bone mineral density (BMD) and bone mineral content (BMC) were obtained using dual energy X-ray absorptiometry (Lunar IDXA, GE Healthcare Madison, WI) at T3.5 (*N* = 98). All scans used the same positioning technique and were analyzed by a single operator using Encore version 16 software. All BMD and BMC results were compared to the GE Lunar internal reference database to generate and report age specific z-scores. Parents reported their child’s fracture history.

#### Psychosocial outcomes

##### Social and emotional skills (T2)

The BarOn Emotional Quotient Inventory-Youth (EQi-YV; short version) assessed social and emotional skills (*n* = 108; T2). The instrument has acceptable validity and reliability [34]. The 30-item EQi-YV, contributing to a total score for emotional intelligence, was administered individually to children. Response options were on a 4-point Likert scale (“not true of me” to “very much true of me”). Total scores were standardised (μ = 100, SD = 15). Higher scores represent more favourable outcomes.

##### Quality of life (T3)

At T3, parents completed the 15-item Pediatric Quality of Life Inventory (PedsQL), reporting their child’s problems with physical, emotional, social and school functioning on a 5-point Likert scale (“never” to “almost always”). Scores were transformed to a 0–100 scale; higher scores indicated more favourable outcomes. The total score was used in analyses. The PedsQL has established feasibility, validity and reliability [35].

##### Self-esteem (T3)

The Harter’s Self-Perception Profile for Children (SPPC) [36] assessed global self-worth and scholastic, social, and athletic competence (T3). Children responded to 24 items by indicating with which of two statements they identified most (e.g. “some kids often *forget* what they learn” or “other kids can remember things *easily*”) and then stating if the statement is “sort of true for me” or “really true for me”. Items are scored on a 1–4 scale. Items within subscales were summed; higher scores indicate more favourable outcomes. The SPPC has established reliability and validity [36].

##### Strengths and difficulties (T3)

Parents completed the Strengths and Difficulties Questionnaire (SDQ; T3), a brief behavioural screening tool with items on 25 positive and negative attributes [37, 38]. Items are scored on a 3-point Likert scale (‘not true’ to ‘certainly true’). Two summary scales, total difficulties and prosocial behaviour, were used as outcomes in analyses. Responses are reverse-scored where necessary and summed. The total difficulties score ranges from 0 to 40 (higher scores indicate more difficulties); prosocial scores range from 0 to 10 (higher scores represent more prosocial behaviour) [39]. Multiple studies support reliability and validity of the SDQ [40].

#### Educational outcomes

##### NAPLAN (age 8–9 years)

Academic achievement was assessed by data linkage (with parent consent) with Year 3 (age approximately 8–9 years) results from the standardised National Assessment Program – Literacy and Numeracy (NAPLAN) [41]. NAPLAN has established reliability and validity [42]. These data were attained from the Victorian Curriculum and Assessment Authority. Achievement is assessed across five domains: language (grammar, punctuation), reading, writing, spelling and numeracy. An achievement score is calculated for each domain based on the number of correct items and then converted to a scale score (0–1000, higher scores indicate greater achievement).

### Confounders (T1)

Child age at T1 (calculated from parent-reported child date of birth), sex, and maternal education (proxy for socioeconomic position) were included in each model as confounders. BMI-z scores (T1) were included as confounders in analyses with BMI-z scores as outcomes. No other outcome variables were assessed at T1. Sex and age standardized z-scores for height (T1) were included as confounders where BMD and BMC variables were outcomes.

### Statistical analyses

Children were categorised at T1 as meeting/not meeting: 1) each individual guideline (for Aims 1 and 2); and 2) all guidelines (for Aim 3) at T1. Descriptive statistics for compliance, outcomes and confounders, were calculated. Multilevel, mixed effect, multivariable linear regression models were used to assess outcomes with normal, continuous distributions. Only 2 outcome variables, the SDQ prosocial score (ordered categorical) and history of fractures (dichotomous), did not meet assumptions for linear regression. Analyses for these outcome variables used ordered logistic regression and multilevel, mixed effects multivariable logistic regression, respectively. Analyses (in Stata SE 15.0) controlled for baseline confounders and clustering by center of recruitment [43]. Associations were examined for compliance with: 1) all guidelines (Aim 3; Model 1); 2) each guideline independently (Aim 1; Model 2); and 3) each guideline when adjusting for compliance with each of the other guidelines (Aim 2; Model 3) with each outcome variable in separate analyses. Complete case analyses were undertaken for each model.

## Results

Children were 4.6 years old (SD = 0.70) at baseline, 53% were boys and 62% were from high, 31% from mid and 7% from low socio-economic families as indicated by maternal education. Most children met PA (89%) and sleep (93%) guidelines; only 23% met the screen-time guideline. Overall, 20% of children met all 3 guidelines. (See Supplementary Table 1 for T1 descriptive statistics.)

Regression analyses results for physiological outcomes are presented in Table 1. Meeting all 3 guidelines at T1 was inversely associated with BMI z-score at T3 (b = − 0.26, 95%CI -0.47, − 0.05). At T3.5, meeting the PA guideline was positively associated with total body BMD and with total body BMC, independent of meeting each of the other guidelines.

**Table 1:** Associations between compliance with Australian 24-Hour Movement Guidelines and physiological outcomes

	Adiposity								Bone Health (age 10–12 years)									
	Waist circumference (age 6–8 years; cm)		Waist circumference (age 9–11 years; cm)		BMI z-score (age 6–8 years)		BMI z-score (age 9–11 years)		Total body BMD z-score		Lumbar Spine (L2–4) z-Score		Total body BMC		Lumbar spine (L2–4)		Fracture history	
	b	95%CI	b	95%CI	b	95%CI	b	95%CI	B	95%CI	b	95%CI	B	95%CI	B	95%CI	OR	95%CI
**Model 1:** Meets all 3 guidelines	−0.33	−1.75, 1.08	−2.01	−4.03, 0.00	− 0.07	− 0.22, 0.07	**−0.26**	**− 0.47, − 0.05**	0.09	−0.32, 0.51	− 0.14	−0.68, 0.41	26.52	−79.32, 132.35	−0.60	−4.32, 3.11	1.26	0.39, 4.07
Model 2: Meets guidelines assessed individually																		
Meets physical activity guidelines	0.79	−1.02, 2.60	0.03	−2.68, 2.73	0.10	−0.09, 0.29	0.08	−0.20, 0.36	**0.67**	**0.23, 1.10**	0.52	−0.07, 1.11	**178.89**	**67.25, 290.54**	3.01	−1.07, 7.10	2.59	0.50, 13.57
Meets screen-time guidelines	−0.20	−1.40, 1.10	− 0.66	−2.39, 1.06	−0.04	− 0.17, 0.08	−0.04	− 0.27, 0.19	−0.07	− 0.45, 0.31	−0.35	− 0.85, 0.14	− 11.28	− 110.11, 87.54	−1.54	−4.95, 1.87	0.90	0.29, 2.79
Meets sleep guidelines	−1.71	− 3.60, 0.18	− 0.81	− 3.59, 1.97	− 0.00	− 0.20, 0.20	−0.04	− 0.34, 0.26	a		a		a		a		a	
Model 3: Meets individual guidelines adjusting for compliance with other guidelines																		
Physical activity	0.83	−0.98, 2.63	0.06	−2.4, 2.76	0.10	−0.08, 0.29	0.13	−0.22, 0.48	**0.67**	**0.23, 1.11**	0.55	−0.04, 1.14	**183.19**	**69.92, 296.46**	3.20	−0.89, 7.30	2.59	0.50, 13.67
Screen-time	−0.34	−1.67, 0.99	−1.56	−3.47, 0.36	−0.09	− 0.22, 0.05	−0.13	− 0.38, 0.12	−0.11	− 0.47, 0.26	−0.38	− 0.87, 0.11	−28.35	−122.00, 65.30	− 1.77	−5.19, 1.65	0.88	0.28, 2.82
Sleep	−1.94	−4.35, 0.47	−1.30	−4.92, 2.33	−0.09	−0.33, 0.16	− 0.36	−0.84, 0.11	a		a		a		a		a	

*BMI* Body mass index, *BMC* Bone mineral content, *BMD* Bone mineral density, *L2–4* Lumbar vertebral spine 2–4, *B*: Regression coefficient, *95%CI* 95% confidence interval; ^a^: compliance with the sleep guideline was omitted from some analyses as all children in the sample were compliant; bolded values are significant at *p* < 0.05

No associations were evident between compliance and psychosocial outcomes (Table 2). Results of regression analyses for educational outcomes are presented in Table 3. Meeting sleep guidelines was associated with higher reading (b = 37.60, 95%CI 6.74, 68.46), spelling (b = 34.95, 95%CI 6.65, 63.25), numeracy (b = 39.09, 95%CI 11.75, 66.44) and language (b = 44.31, 95%CI 11.77, 76.85) scores at 8–9 years of age when examined in individual models (Model 2). When associations were adjusted for compliance with other guidelines, results were similar and compliance with the sleep guideline was also associated with writing achievement (b = 25.93, 95%CI 0.30, 51.57). No associations with educational outcomes were evident for meeting either PA or screen-time guidelines.

**Table 2:** Associations between compliance with Australian 24-Hour Movement Guidelines and psychosocial outcomes

	Bar-On EQi-Yv		Peds HRQoL				SPPC								SDQ			
	Emotional skills scores		Psychosocial scores		Total score		Scholastic competence		Social competence		Athletic competence		Global self-worth		Prosocial		Total problem behaviors	
	B	95%CI	B	95%CI	B	95%CI	B	95%CI	B	95%CI	B	95%CI	B	95%CI	OR	95%CI	B	95%CI
Model 1: Meets all 3 guidelines	−3.99	−11.97, 3.99	0.00	−3.30, 3.31	−0.14	− 3.16, 2.88	−0.07	−0.22, 0.09	− 0.03	− 0.18, 0.11	0.02	− 0.13, 0.16	− 0.09	−0.22, 0.04	1.00	0.64, 1.54	−0.08	−1.31, 1.16
Model 2: Meets guidelines assessed individually																		
Meets physical activity guidelines	−1.83	−10.44, 6.78	0.81	−3.74, 5.36	0.99	−3.17, 5.15	−0.11	− 0.33, 0.10	0.02	− 0.17, 0.21	0.11	− 0.09, 0.31	−0.13	− 0.31, 0.04	0.98	0.57, 1.70	−0.00	−1.70, 1.70
Meets screen-time guidelines	−0.25	−6.51, 6.00	0.20	−2.66, 3.06	0.07	−2.62, 2.75	−0.14	−0.28, 0.00	− 0.02	−0.15, 0.11	0.06	−0.07, 0.20	− 0.09	−0.20, 0.03	1.01	0.70, 1.46	0.04	−1.01, 1.09
Meets sleep guidelines	−3.11	−12.41, 6.19	1.52	−3.25, 6.28	2.34	−2.15, 6.83	0.19	−0.04, 0.42	−0.16	− 0.38, 0.05	0.02	− 0.20, 0.23	0.01	− 0.18, 0.21	0.83	0.50, 1.37	−0.36	− 0.21, 1.38
Model 3: Meets individual guidelines adjusting for compliance with other guidelines																		
Physical activity	−1.91	−10.56, 6.73	0.80	−3.76, 5.35	0.99	−3.16, 5.15	−0.11	−0.32, 0.10	0.02	−0.17, 0.21	0.11	−0.09, 0.31	− 0.13	−0.31, 0.05	0.98	0.57, 1.69	0.01	−1.69, 1.70
Screen-time	−0.14	−7.76, 7.48	0.18	−3.00, 3.36	−0.04	−2.94, 2.87	−0.09	− 0.24, 0.06	−0.00	− 0.14, 0.13	0.05	− 0.09, 0.19	−0.06	− 0.18, 0.07	1.01	0.70, 1.51	−0.15	−1.34, 1.04
Sleep	1.71	−11.21, 14.63	3.14	−2.92, 9.20	4.08	−1.45, 9.61	.020	−0.09, 0.48	−0.10	− 0.36, 0.16	0.08	− 0.19, 0.35	0.13	− 0.12, 0.37	0.85	0.48, 1.52	−0.59	−2.85, 1.67

*EQi-YV* Emotional quotient inventory, youth version, *PedsQL* Pediatric Quality of Life, *SPPC* Harter’s Self Perception Profile, *SDQ* Strengths and Difficulties Questionnaire, *B* Regression coefficient, *95%CI* 95% confidence interval

**Table 3:** Associations between compliance with Australian 24-Hour Movement Guidelines and educational outcomes

	Reading		Writing		Spelling		Numeracy		Language (grammar & punctuation)	
	B	95%CI	B	95%CI	B	95%CI	B	95%CI	B	95%CI
**Model 1:** Meets all 3 guidelines	6.28	−15.66, 28.21	9.26	−5.67, 24.20	12.80	−7.34, 32.94	6.31	−13.07, 25.69	9.49	−14.01, 32.99
Model 2: Meets individual guidelines assessed individually										
Meets physical activity guidelines	−19.20	−49.94, 8.54	−9.20	−28.10, 9.69	−10.98	−36.16, 14.20	− 13.91	−38.20, 10.38	−12.89	−41.3, 15.84
Meets screen-time guidelines	−1.45	−20.67, 17.80	0.24	−12.81, 13.29	−3.75	−21.69, 14.19	−1.41	− 18.51, 15.69	−5.25	−25.97, 15.47
Meets sleep guidelines	**37.60**	**6.74, 68.46**	20.70	−0.08, 41.47	**34.95**	**6.65, 63.25**	**39.09**	**11.75, 66.44**	**44.31**	**11.77, 76.85**
Model 3: Meets individual guidelines adjusting for compliance with other guidelines										
Meets physical activity guidelines	−18.68	−46.03, 8.67	−9.04	−27.83, 9.74	−10.74	−35.59, 14.12	− 13.81	−37.76, 10.14	−12.64	− 41.11, 15.82
Meets screen-time guidelines	2.25	−18.02, 22.52	5.37	−8.56, 19.29	−1.02	−19.59, 17.54	1.20	−16.63, 19.03	0.03	−21.64, 21.71
Meets sleep guidelines	**53.23**	**14.98, 91.48**	**25.93**	**0.30, 51.57**	**56.10**	**22.04, 90.17**	**55.74**	**22.97, 88.50**	**61.07**	**21.41, 100.73**

*B* Regression coefficient, *95%CI* 95% confidence intervals; bolded values are significant at *p* < 0.05

## Discussion

This study examined Australian preschoolers’ compliance with Canadian/Australian 24-Hour movement guidelines and prospective associations with physiological, psychosocial and educational outcomes. Although only 20% of children met all guidelines, most met sleep (93%) and PA (89%) guidelines. Compliance with the sleep guideline was associated with all educational outcomes when children were aged age 8–9 years in adjusted models. Compliance with the PA guideline was associated with total body BMD and BMC when children were aged 11–13 years. Meeting all guidelines was associated with more favourable (i.e., lower) BMI 6 years later (ages 9–11 years). Psychosocial outcomes were not associated with guideline compliance.

The proportion of children complying with the new 24-Hour Movement Guidelines for the Early Years is similar to a recent cross-sectional Australian study in preschoolers [18], but higher than studies in Australian toddlers [44] and Canadian preschoolers and toddlers [19, 45]. Earlier studies are comparable only on screen-time guidelines where estimates suggest compliance between 3 and 63% [46, 47]. Variance between studies may be due to methodological or participant characteristics. For instance, compliance may be assessed by including all screen-time components (e.g. television, computer, electronic games) [23, 48], or combinations thereof (e.g. television, electronic games) [46, 49], or may be measured continuously [23] or in discrete categories [50]. This study captured data on TV/DVD/video viewing, internet/computer use and electronic games. Future studies should consider including all forms of screen-time when estimating compliance and measuring time in behaviors continuously for more sensitive estimates.

A recent systematic review of preschoolers’ PA and a variety of outcomes identified only 10 longitudinal studies and reported associations were inconsistent across studies [10]. Reported associations were evident across shorter time-frames (e.g. 12 months [51–53]) with only three studies showing associations with quality of life [54, 55] and fracture risk [56] over longer periods. Consistent with those studies, the current study found few associations of compliance with PA guidelines during early childhood and later outcomes, with the exception of bone health. Our findings concur with earlier review findings that PA during the pre-pubertal period, from as young as 6 years of age, is important for bone health [57], and extends previous work to highlight the importance of physical activity from early life. Some evidence suggests that the magnitude of the effects found in this study for associations between physical activity and bone health are meaningful [58–60]. Thus, all parents, early childhood educators and practitioners should encourage children to participate in adequate levels of PA as preschoolers.

In the current study, meeting screen-time guidelines during the preschool years was not associated with any of the assessed outcomes. A recent review examining associations between screen-time and health and developmental outcomes reported associations were primarily unfavorable or null [11]. Of the included studies, 19 were longitudinal and reported associations of preschoolers’ screen-time with outcomes 1 to 9 years later. Associations of screen-time with psychosocial outcomes were primarily null, as in the current study, while those with cognitive outcomes were mixed [11]. Differences in findings may be attributable to differences in methodologies, including measurement of screen-time or types of devices used, or where a continuous measure of screen-time was used which would allow for identification of potential dose-response associations [61, 62]. Future studies exploring dose-response associations are required [11]. Research investigating associations between use of different devices and associations with outcomes is also warranted.

Meeting the sleep guideline was consistently and strongly associated with educational outcomes. The current study found differences in NAPLAN scores of up to 61 points between those meeting and not meeting the sleep guideline. This test score difference equates to change of approximately one performance band, or between meeting and not meeting national minimum standards [63, 64]. Previous longitudinal research suggests that shorter sleep duration during early childhood may be associated with poorer outcomes for adiposity, emotion regulation and cognitive outcomes, and not associated with psychosocial outcomes [9]. However, findings between studies were mixed [9]; only 1 prospective study has examined preschoolers’ sleep and later cognitive outcomes [65]. That study found children’s night-time sleep duration at 3 years was associated with cognitive ability and children with higher cognitive ability at 3 years maintained higher abilities over a 3-year period [65]. Other research indicates that preschoolers’ cognitive ability is predictive of academic achievement during primary school [66]. Thus it may be that preschoolers’ sleep is associated with educational achievement, as evident in this study, through early cognitive ability. However, this remains to be tested in future research.

This study also explored associations of compliance with all guidelines. Few studies have previously explored such associations in preschoolers [8, 19, 21, 67], and only three studies have reported prospective associations of behaviours [20, 22] or compliance with guidelines [21] and later outcomes. Those studies found that neither behaviors nor compliance with guidelines were associated with indicators of adiposity across one to four years. Similarly, the current study found no association between compliance with all guidelines at baseline and BMI z-score 3 years later but did find a significant inverse association between compliance and BMI z-score 6 years later. Previous research suggests that the magnitude of this association may be clinically meaningful [68]. It may be that early propensity for healthful behaviors, characterized here by meeting all guidelines, is supportive or indicative of healthful behaviors generally over time, which subsequently manifests in the identified association. Additionally, it may be necessary for children to have prolonged exposure over time to health behaviours for associations with outcomes such as BMI to become evident. Thus, the longer follow-up time in this study may be necessary for associations to be identifiable. It is noteworthy that compliance with the individual guidelines was not associated with BMI over time, despite compliance with all guidelines showing a favourable association with BMI 6 years later. Thus, it is possible that favourable outcomes for some variables, such as BMI, rely on children participating in a range of healthy behaviours.

Findings from this study highlight opportunities for future research. Given the number of null associations identified here, updated guidelines may not accurately identify the most appropriate thresholds for each behavior beneficial for all outcomes. Although current guidelines are based on the best available evidence, reviews undertaken to establish those guidelines highlighted that evidence to identify daily thresholds is insufficient and is of low to moderate quality [2]. Thus research should examine prospective associations between continuous values of each behavior and multiple outcomes to identify thresholds to better inform future guidelines. In particular, strong and consistent associations between compliance with the sleep guideline and children’s educational outcomes warrant further attention. Additionally, behavior at a single time point may not be sufficient to predict future outcomes. Rather, behavior across time points may be a stronger predictor of outcomes. Thus, research should endeavor to establish associations of behaviors across time with later outcomes and future guidelines may reflect this. Characteristics of these behaviors which are not considered in current guidelines may also be important. For instance, future studies should investigate associations of characteristics of behaviours with later outcomes. These may include: type of PA (e.g. individual, structured [10]) or screen-time (e.g. TV, educational apps), caregiver interaction [69–71], patterns [11, 72] (e.g. short, sustained), timing (e.g. screen-time immediately before bed [73]), sleep onset/waking times, and sleep quality.

### Strengths and limitations

This study helps to address gaps recognized in evidence informing the guidelines [9, 10]. Strengths of the study include use of an objective measure of physical activity in a large sample of preschool children. The inclusion of multiple types of electronic media when assessing screen-time is noteworthy, since previous research has focused mainly on TV viewing [11]. Further strengths are the prospective design providing evidence of temporal associations, inclusion of exposure and outcome variables with established psychometric properties, an objective measure of PA and multiple outcomes. In addition to multiple strengths, this study also has some limitations that should be noted. NAPLAN tests provide a point-in-time estimates of children’s literacy and numeracy and are intended for use in combination with other school-based assessment programs not available for inclusion in this study [74]. The small proportion (7%) of children who did not meet the sleep guideline may be qualitatively different from those who did, and may help explain differences in outcomes. However, this is beyond the scope of this study. The response rate was 11% and a self-selected sample. This may mean that the results are not generalizable to the Australian population. However, a large, heterogenous sample of children across multiple sites was recruited. Additionally, the sample was comparable with the Australian population on important demographic characteristics. For instance, when characteristics of this sample are compared with the Australian population, 70% of parents in this sample were born in Australia as were 70% of parents in the total population, 67% vs 58% of parents have post-secondary qualifications, and 88% vs 78% of families are dual-parent, respectively [75]. Thus, these findings are likely to be generalizable to children in Australia. While we recruited from centres across socioeconomic strata, only a small proportion of the final sample represented this demographic. Data from 2 of the exposure variables, and most of the outcome variables, are subjective; results may vary if more objective data are available. Not all outcomes were assessed at all time points due to evolution in the focus of the study. While this limits the ability to directly compare between outcome measures, it adds breadth to the range of outcomes that can be commented on in this study.

## Conclusion

This is the first study to examine longitudinal associations between meeting the integrated movement guidelines during preschool and health and developmental outcomes. It found consistent associations between compliance with PA guidelines during early childhood and future bone health, and between compliance with sleep guidelines during early childhood and later educational outcomes. This study highlights the benefits of meeting guidelines during early childhood which enables children to maximise their later health and educational potential.

## Data Availability

The dataset supporting the conclusions of this article is available on request from the authors pending ethical clearance.
